# Place and Space: How does the hospital environment influence the inclusion of care partners of hospitalized people living with dementia?

**DOI:** 10.1002/alz.089502

**Published:** 2025-01-09

**Authors:** Catherine Still, Sydney Hoel, Andrea Strayer, Teresa Thuemling, Nicole Werner, Beth Fields

**Affiliations:** ^1^ University of Wisconsin‐Madison, Madison, WI USA; ^2^ Indiana University, Bloomington, IN USA; ^3^ University of Iowa, Iowa City, IA USA

## Abstract

**Background:**

Care partners of hospitalized people living with dementia (PLWD) are particularly vulnerable in the hospital setting and may feel invisible during hospitalization of the PLWD they care for. Hospital initiatives for dementia friendly spaces emphasize providing a safe and supportive environment. However, literature is scarce regarding how the hospital’s physical environment influences care partners' inclusion. Therefore, we aimed to explore how the physical environment influences care partners’ inclusion in hospital‐based dementia care.

**Method:**

Using a descriptive qualitative design and a multi‐methods approach to data collection, our team conducted direct observations in a large academic hospital’s trauma/medical surgical unit, orthopedic unit, family practice unit, and neuroscience/stroke unit. We also interviewed care partners and clinicians to gain insight into the impact the hospital environment has on the inclusion of care partners. Observational data were analyzed using a well‐known systems engineering framework, and interview data were analyzed thematically.

**Result:**

Direct observation (27 hours), care partner (15) and clinicians interviews (23) yielded insights into underutilized physical environment elements in the hospital and opportunities to enhance care partner inclusion through environmental modification. Underutilized physical environment elements included family‐designated spaces and communication tools like caregiving education bulletin boards and patient whiteboards. Care partner inclusion could be enhanced by having enough space for the care partner to be in the patient’s room comfortably, increasing opportunity to personalize spaces, and making parking more accessible.

**Conclusion:**

Our findings demonstrate a need to augment the physical environment of the hospital setting. To facilitate the inclusion of care partners to PLWD, health systems must consider ways to improve their physical environment to increase care partners’ comfort and accessibility. Enhancing the physical environment of the hospital setting has potential to increase the satisfaction, health, and well‐being of both PLWD and their care partners.

